# Equity, evidence and Indigenous knowledges in PROMs in Australian injury research: a systematic review

**DOI:** 10.1007/s11136-026-04353-2

**Published:** 2026-07-30

**Authors:** Georga Sallows, Patrick Sharpe, Pip Henderson, Lavender Otieno, Daniel Y. Ellis, Nicole Kelly, Mahni Foster, Belinda Gabbe, Mark Fitzgerald, Julieann Coombes, Luke Wolfenden, Brett Shannon, Gerard O’Reilly, Murthy Mittinty, Kate Hunter, Shanti Omodei-James, Hossein Haji Ali Afzali, Maree Toombs, Courtney Ryder

**Affiliations:** 1 Collage of Medicine and Public Health, Flinders University, Adelaide, SA Australia; 2 Flinders Health and Medical Research Institute, Flinders University, Adelaide, SA Australia; 3 Engage, Motivate, Unite (E.M.U) Consultancy, Ceduna, SA Australia; 4 Trauma Service, Royal Adelaide Hospital, Adelaide, SA Australia; 5 Faculty of Health and Medical Sciences, University of Adelaide, Adelaide, SA Australia; 6 School of Public Health and Tropical Medicine, James Cook University, Cairns, QLD Australia; 7 Department of Epidemiology and Preventive Medicine, Monash University, Melbourne, Australia; 8 National Trauma Research Institute, The Alfred Hospital, Melbourne, Australia; 9 Department of Surgery, Central Clinical School, Monash University, Melbourne, Australia; 10 Trauma Service, Alfred Health, Melbourne, Australia; 11 The George Institute for Global Health, Sydney, NSW Australia; 12 School of Population Health, University of New South Wales, Sydney, NSW Australia; 13 School of Medicine and Public Health, The University of Newcastle, Newcastle, Australia; 14 School of Public Health, University of Illinois Chicago, Chicago, IL USA

**Keywords:** Aboriginal and Torres Strait Islander, Patient reported outcome measures, PROMs, Injury, Equity, HRQoL

## Abstract

**Purpose:**

Patient-reported outcome measures (PROMs) are widely used in trauma and injury research to assess health-related quality of life (HRQoL), mental health and functional recovery. However, PROMs are rarely adapted to reflect the lived experiences, cultural values and determinants of health for priority populations, particularly Aboriginal and Torres Strait Islander peoples, who experience a disproportionate burden of injury in Australia. This systematic review addressed the inclusion of health equity indicators and Indigenous knowledges for PROMs used in observational Australian trauma and injury studies for adults (≥ 18 years).

**Methods:**

A systematic search of Medline (OVID), CINAHL (EBSCO) and Scopus was performed including prospective cohort or longitudinal designs, focused on trauma-related injuries conducted in Australia. Data were extracted and appraised using the CREATE and PROGRESS-PLUS tools to assess Indigenous engagement and equity-sensitive reporting.

**Results:**

Across the 11 included studies, 17 different PROMs were used with SF-36 and PCL-C being the most common. Physical and mental health outcomes were consistently impaired post-injury, with long-term impacts. Equity considerations were largely absent; none of the studies met all PROGRESS-PLUS criteria and Indigenous knowledges were not incorporated. Priority populations, including Aboriginal and Torres Strait Islander peoples, culturally and linguistically diverse (CALD) communities, people with disabilities and women, were frequently underrepresented or excluded.

**Conclusion:**

These findings highlight significant gaps in equity-sensitive PROM use and the urgent need for culturally safe and inclusive PROM development that reflects diverse recovery experiences thereby supporting equitable trauma care and follow-up.

**Supplementary Information:**

The online version contains supplementary material available at https://doi.org/10.1007/s11136-026-04353-2.

## Abstract (plain language summary)

This study looked at how injury research in Australia uses patient-reported outcome measures (PROMs) to understand recovery after injury. PROMs are tools that ask patients about their health and wellbeing, but they often don’t reflect the experiences or cultural values of Aboriginal and Torres Strait Islander peoples, who are more likely to be injured and face worse outcomes. We reviewed 11 studies and found that while 17 different PROMs were used, none were designed with or for Aboriginal and Torres Strait Islander communities. Most studies didn’t include key equity factors like race, language, disability, or gender in their analysis, and Indigenous perspectives were missing entirely. This means current trauma research may not correctly capture the recovery journeys of priority populations. Our findings show an urgent need to develop culturally safe and inclusive PROMs that reflect diverse experiences and support fairer trauma care. This work can help improve health outcomes and ensure that Aboriginal and Torres Strait Islander peoples are meaningfully included in future research and healthcare planning.

**Supplementary Information:**

The online version contains supplementary material available at https://doi.org/10.1007/s11136-026-04353-2.

## Introduction

Patient reported outcome measures (PROMs) are important tools used to capture an individual’s own assessment of health-related quality of life (HRQoL), functional outcomes, physical health, mental health and wellbeing [1]. HRQoL tools such as PROMs, are commonly used in trauma and injury (from here on referred to as injury) to capture patient perspectives on the impact of the initial injury as well as the journey to recovery through treatment and rehabilitation [2, 3]. Despite their common use in clinical and research environments, there is growing concern that PROMs may not adequately reflect the lived experiences, cultural values, or cultural determinants of health relevant to priority populations, including Aboriginal and Torres Strait Islander peoples [4].

In Australia, Aboriginal and Torres Strait Islander peoples are the First Nations peoples. Since time immemorial, they have established and evolved sophisticated holistic approaches to knowledge, innovation, health and wellbeing, reflected in being the first philosophers, researchers, educators, curators, engineers and healthcare practitioners on the continent. Despite this, Aboriginal and Torres Strait Islander communities face a disproportionate disease and injury burden compared to the dominant Australian population [5, 6], contributing to health inequities directly linked to ongoing colonisation. Injury impacts are of particular concern, as the third leading cause of death and second leading cause of disease burden for Aboriginal and Torres Strait Islander communities, with elevated rates across self-harm, burns [7], road traffic crashes [8], poisoning and suicide [9–12]. In Aboriginal and Torres Strait Islander adults (≥ 18 years) the highest rates of hospitalisation occur across 25–44 years of age, significantly impacting important labour-force years for these individuals and their communities [13, 14]. Aboriginal and Torres Strait Islander people are often discharged from hospital against medical advice at significantly higher rates than non-Indigenous people [15], largely due to factors such as cultural insensitivity, racism, mistrust of the health system, language barriers, and feelings of fear or isolation [16]. These health inequities highlight the urgent need for culturally relational measures and indicators of care for Aboriginal and Torres Strait Islander communities to combat this disproportionate burden of injury [17, 18]. Measuring PROMs across any population group is inherently complex due to the multidimensional nature of health and wellbeing and inconsistent definitions across disciplines. For these reasons, PROM design in Australia focuses on the dominant population, utilising Western biomedical constructs and knowledge for creation and assessment [4, 19–21]. In PROM psychometric assessment, face and content validation involving end-users is considered best practice, particularly for cultural appropriateness of items [22], however, these methods, particularly those including consumers or community perspectives, are among the least used despite their importance in preventing ethnocentric bias and invalid factor constructs [22].

Australia has clear national guidelines and frameworks surrounding Aboriginal and Torres Strait Islander research that emphasise the need for community leadership, self-determination, sustainability, impact and accountability to community [4, 21, 23, 24]. Despite this, research involving PROMs with Aboriginal and Torres Strait Islander communities rarely uses tools which have been psychometrically assessed and validated in these populations [20, 25]. These approaches and PROMs fail to reflect Indigenous knowledge, worldviews, values and relationships, including connections to community, Country,^1^ spirituality and culture, all of which are central to health and wellbeing [26]. This is further reflected in lower PROM completion rates by Aboriginal and Torres Strait Islander participants [27].

In the Australian injury research landscape, it currently remains unknown if existing PROMs have been developed in relational ways, which centre Indigenous knowledges (knowing, being and doing) and approaches to health and wellbeing. The aim of this systematic review is to comprehensively examine observational injury studies in Australia that have employed PROMs and assess and critique how these studies incorporate considerations of health equity and Indigenous knowledges.

## Methods

### Search strategy and selection criteria

Search terms, including MeSH, were developed by authors GS and CR from key literature and consultation with clinicians, researchers, Aboriginal consumer investigators and a specialist librarian, resulting in syntax and terms outlined in Table 1. Database searches of Medline (OVID), CINAHL (EBSCO) and Scopus were conducted in late March 2025, covering literature published from January 2000 to March 2025.

**Table 1:** Example of search syntax used in one of the data bases

Databases	Example Syntax (Scopus)
Medline (OVID), CINAHL (EBSCO) and Scopus	1. "Australia*" OR "United States of America" OR "the US" OR "USA" OR "Canada" OR "New Zealand" OR "Aotearoa" 2. hospital* OR inpatient* OR (tertiary OR secondary OR acute) W/1 care)) OR ("Accident and emergency" OR "A & E" OR "Emergency Room" OR er OR "Emergency Department" OR casualty OR triage OR "Emergency Ward" OR "Emergency Medicine" OR surgical) 3. injur* OR accident* OR unintentional OR traffic OR drown* OR poison* OR burn* OR fall* OR intentional OR "physical violence" OR "mental violence" OR abuse OR neglect OR "negligent treatment*" OR maltreatment* OR suspicious 4. "Patient-Reported Outcome*" OR prom OR "self-report* outcome*" OR ( patient W/2 ( recorded OR reported OR reporting OR based OR centered) W/2 outcome*) OR ( "Health Related Quality of Life" OR "Quality of Life" OR qol OR "Health Status" OR "Activities of Daily Living" OR "Glasgow Outcome Scale") OR ( "EQ-5D" OR hqol OR "SF-12" OR mqol OR qwb OR boq OR "SF-36" OR whodas OR ffs OR hrqol) 5. adolescent* OR child* OR infant* OR preschool* OR p?ediatric 6. 1 AND 2 AND 3 AND 4 AND NOT 5 7. limit 6 to (English language and yr = ‘2000 -Current’)

Initially high-income countries with a shared colonial history, the United States of America, Canada, New Zealand and Australia, were included to capture a large number of studies in post-colonial settings that are now characterised by substantial cultural, ethnic and linguistic diversity. The search strategy was intentionally broad and did not restrict studies by Indigenous status, as First Nations peoples are frequently included within broader population-based injury studies rather than being reported separately. Following full-text review, the scope of this review was refined to focus specifically on the Australian context. Studies conducted outside Australia (n = 5) were therefore excluded to enable a more detailed examination of patient-reported outcomes relevant to Australian healthcare systems and Aboriginal and Torres Strait Islander peoples. Key inclusion and exclusion criteria were created by authors GS and CR to capture PROMs for adults (≥ 18 years), from observational (cohort, longitudinal, follow-up and prospective) studies (Table 2). Following guidelines on injury outcome research, all studies needed to include ≥ 3 time points, which included a baseline measurement before discharge from hospital, rehabilitation/adaptive phase 1–12 months and stable 6–24 months, time points [28, 29].

**Table 2:** Selection criteria for articles

Inclusion	Exclusion
All papers focused on injury	Non-injury related studies
Participants ≥ 18 years old	Studies reporting on populations < 18 years were excluded for analysis
Observational studies	Non-observational studies, retrospective studies or case studies
All studies were required to have at least three time points, with baseline occurring pre-hospital discharge, and two further follow up time points	Studies focussed on: study protocols, clinical treatment, conference abstracts, clinical guidelines, rehabilitation outcomes and other guidelines
Articles were restricted to published findings in English-language only over the last 25 years (January 2000 – March 2025)	Studies assessing the psychometric properties of PROMs
At the full text phase	
Studies focusing on populations living in Australia	All other countries

### Data extraction

The review followed PRISMA-E 2012 guidelines, an established framework specific to equity-focussed systematic reviews [30], and the CONSIDER statement for reporting of health research involving Aboriginal and Torres Strait Islander peoples [31]. A two-step search strategy was conducted. Title and abstract screening against key words and inclusion and exclusion criteria was undertaken by the lead author (GS) in Covidence. Quality assurance was undertaken at the screening phase through a ‘blinded review’ process, where an independent reviewer (PH) assessed 20% of the papers. The results from this blinded review were 99.6% consistent with that of GS. Conflicts were discussed and resolved with guidance from CR where appropriate.

Data extracted from included articles into Microsoft Excel comprised of reference, study location, aim, ethics approval, study type, setting, injury focus, sociodemographic results, PROM, study duration, time points (follow up), participant numbers (baseline, follow up), key findings, strengths and limitations.

### Quality appraisal

Study methodology, design and outcome reporting which targeted equity and Indigenous knowledge was undertaken using two tools (CREATE, PROGRESS-PLUS) to extract data under three domains:

1. Demographics: reference, study type, injury focus, study location, PROM used, data collection times; Indigenous knowledges and community engagement
2. CREATE tool: Aboriginal leadership, methodology and methods, community consultation and governance and strength-based analyses and [32, 33]
3. Health Equity (PROGRESS-Plus): residence, race/ethnicity, occupation, gender, religion, education, social-capital, socioeconomic position, age, disability, sexual orientation, other vulnerable groups [30, 34, 35].

### Narrative synthesis

A narrative-based synthesis was used to comprehensively critique and describe evidence on injury PROMs and their design for Aboriginal and Torres Strait Islander communities. This systematic review followed the three-step process outlined by Popay et al. [36] and Petticrew and Roberts [37].

### Risk of bias

Risk of bias was evaluated using the RTI Item Bank (Research Triangle Institute, California, USA) [38], a tool specifically developed to assess bias and precision in observational studies. It comprises 29 questions across 11 domains, ranging from sample definition to outcome analysis. For this systematic review, the following domains were considered: sample definition and selection, exposure assessment, data reliability, follow-up procedures, analytical comparability, outcome analysis, and interpretation.

## Results

### Search results

The electronic database search resulted in a total of 4,118 records and additional two records were identified from reference lists. Duplication removal resulted in 3,064 for review. Title and abstract screening removed 2,965 records, leaving 99 articles for full text review. Full text review of the 99 articles resulted in 11 articles being included for full data extraction (See PRISMA diagram in Appendix 1).

### Demographics

Across the included studies, most were prospective cohort designs conducted in metropolitan trauma centres, primarily in South-East Queensland. Burns were the most studied injury, and a wide range of PROMs were used, with no consistent measurement approach across studies. Follow-up periods varied, although all studies included at least three time points. Overall, substantial variability was observed in study characteristics and outcome reporting, see Table 3. The majority of studies were prospective cohort studies (n = 8, 81.1%) [39–47], followed by longitudinal prospective studies (n = 2, 18.2%) [48, 49]. All studies were conducted in Australia, with focus in South-East Queensland (n = 6, 54.5%) [39–42, 48, 49], followed by Sydney [45, 46] (n = 2, 18.2%) and one each in Melbourne [43], Perth [44], and Canberra [47]. All studies recruited from major trauma centres in metropolitan areas. Recruitment settings varied, with most being a single hospital (n = 7, 63.6%) [40–42, 44, 45, 48, 49], followed by two hospitals (n = 3, 27.3%) [39, 46, 47], with one study recruiting from three hospitals (9.1%) [43]. Four of the 11 studies had multiple injury focuses (36%) [39–42], and the remainder focused on one injury type [43–49]. Burns were the most frequently examined injury type (n = 7, 63.6%) [39, 40, 42, 44, 45, 48, 49], followed by blunt force trauma (n = 4, 36.4%) [39–42], poisonings [39, 40, 42], drownings [39, 40, 42] and road traffic injuries [41, 46, 47] (each n = 3, 27.3%). Other injuries included falls [41], physical violence [41], recreational injury [41] and general traumatic injury [43] (each n = 1, 9.1%). All studies reported at least three data collection points with baseline measurement at hospital discharge and follow up at six months post-injury (n = 11, 100%). Other follow up time points included; one month (n = 4, 36.4%) [40–42, 44], six weeks (n = 1, 9.1%) [43], three months (n = 6, 54.5%) [39, 43–45, 48, 49], 12 months (n = 8, 72.7%) [41, 43–49] and 24 months (n = 3, 27.3%) [41, 45, 46]. Reporting on ethics body approval and the approval number was provided in four (36.4%) of the studies [41, 44, 48, 49], with three reporting just the ethics body (27.3%) [43, 46, 47], while three other studies stated ethics approval no evidence (number/board) was provided [39, 40, 42] and in one study no ethics approval or a statement was provided [45].

**Table 3:** Data items and quality appraisal of included studies

							PROGRESS-Plus** ✓-reported, X -not reported, R –recorded^***^ E -excluded											
						CREATE*									PLUS			
First author, year	Study type	Injury	Location	Outcome measure	Follow up	> 1	P	R	O	G	R	E	S	S	1	2	3	4
Aitken [39]	Prospective Cohort	Burns, poisonings, drownings, blunt trauma	South-East QLD	SF-36v2, TSCS, IASS, RIPQ	Discharge, 3 & 6 months	X	X	X	✓	✓	X	✓	✓	X	✓	E	X	E
Aitken [37]	Prospective Cohort	Burns, poisonings, drownings, blunt trauma	South-East QLD	SF-36v2, SES, BIPQ, MSPSS, K10, PCLC	Discharge, 1 & 6 months	X	X	✓	✓	✓	X	✓	✓	X	✓	E	X	E
Aitken [38]	Prospective Cohort	Road traffic crash, falls, physical violence, blunt trauma, recreational trauma	South-East QLD	SF-36v2, SES, BIPQ, MSPSS, K10, PCLC	Discharge, 1, 6, 12, & 24 months	X	X	X	✓	✓	X	✓	✓	X	✓	E	X	E
Connolly [39]	Prospective Cohort	Burns, poisonings, drownings, blunt trauma	South-East QLD	SES, BIPQ, MSPSS, K10, PCL-C	Discharge, 1 & 6 months	X	X	E	✓	✓	X	X	✓	✓	✓	E	X	E
Clay [40]	Prospective Cohort	Traumatic injury	VIC	SF-36, AQoL, RtW	Discharge, 1.5, 3, 6 & 12 months	X	X	E	✓	✓	X	✓	X	✓	✓	E	X	X
Connell [41]	Prospective Cohort	Burns	Perth	BSHS-B	Discharge, 1, 3, 6 & 12 months	X	X	X	X	✓	X	X	X	X	✓	X	X	X
Dowda [42]	Prospective Cohort	Burns	Sydney	BSHS-B	Discharge, 3, 6 & 12 months	X	X	E	✓	✓	X	X	X	X	✓	E	X	E
Druery [45]	Longitudinal Prospective	Burns	South-East QLD	BSHS-B, SF-12, RtW, MSPSS	Discharge, 3, 6 & 12 months	X	✓	✓	✓	✓	X	✓	✓	✓	✓	E	X	E
Ford [46]	Longitudina Prospective	Burns	South-East QLD	PHQ-9, PCL-C	Discharge, 3, 6 & 12 months	X	✓	R	R	R	X	R	✓	R	✓	E	X	E
Littleton [44]	Prospective Cohort	Road traffic crash	Canberra	SF-36, HADS, FRI	Discharge, 6 & 12 months	X	X	E	✓	✓	X	✓	✓	X	✓	X	X	E
Murgatroyd [43]	Prospective Cohort	Road traffic crash	Sydney	SF-36v2, PCL-C, GRC	Discharge, 6 & 12 months	X	X	✓	✓	✓	X	✓	✓	X	✓	E	X	E

Key: QLD = Queensland, SF-36 = Short Form 36, SF-36v2 = Short Form 36 version 2, TSCS = Therapeutic Self-Care Scale, IASS = Information, Autonomy and Support Scale, RIPQ = Revised Illness Perception Questionnaire, MSPSS = The Multidimensional Scale of Perceived Social Support Questionnaire, PCL-C = The PTSD Checklist Civilian Version, K10 = The Kessler Psychological Distress Scale, SES = Self-Efficacy Scale, BIPQ = The Brief Illness Perception Questionnaire, RtW = Return to Work, AQoL = Assessment of the Quality of Life Instrument, BSHS-B = Burn Specific Health Scale – Brief Version, SF-12 = Short Form 12, PHQ-9 = Patient Health Questionnaire, HADS = Hospital Anxiety and Depression Scale, FRI = Functional Rating Index, GRC = Global Rating of Change

*CREATE Tool: At least one of the CREATE criteria was met by the study

**PROGRESS-Plus: P = Place of residence, R = race/ethnicity/language, O = occupation, G = gender, R = religion, E = education, S = social capital, S = socio-economic position (SEP), 1 = age, 2 = disability, 3 = sexual orientation, 4 = other vulnerable groups

***Recorded but not reported: the study recorded race/ethnicity/culture/language. Occupation, gender, education, and socioeconomic position and analysed in the initial univariate analysis, but excluded these demographics for the final multivariate analysis

### Outcome measures

The majority (n = 9, 82%) [39–43, 46–49] of studies included more than one PROM over each of the reporting time points. There was no consistent pattern in the number of PROMs used across studies or time points. Three studies used three PROMs each [43, 46, 47], while others used varying numbers with one [44, 45], four [39, 48], and up to six [40, 41] PROMs being used across two studies with up to two [49] and five PROMs [42] used in another study. A total of 17 different PROMs were used across the 11 studies.

The most reported PROM was the Short Form-36 including version 2 in over half of the studies (n = 6, 63.6%) [39–41, 43, 46–48]. The second most frequently reported PROM in 45.5% (n = 5) of studies was the Posttraumatic Stress Disorder Checklist [40–42, 46, 49], followed by the Multidimensional Scale of Perceived Social Support (n = 4, 36.4%) [40–42, 48], Self-Efficacy Scale (n = 3, 27.3%) [40–42], the Brief Illness Perception Questionnaire (n = 3, 27.3%) [40–42], the Kessler Psychological Distress Scale (n = 3, 27.3%) [40–42], and the Burn Specific Health Scale–Brief (n = 3, 27.3%) [44, 45, 48]. Less frequently used PROMs included return-to-work measures (n = 2, 18.2%) [43, 48], the Assessment of Quality of Life (n = 1, 9.1%) [43], Short Form 12 (n = 1, 9.1%) [48], the Patient Health Questionnaire (n = 1, 9.1%) [49], the Hospital Anxiety and Depression Scale (n = 1, 9.1%) [47], Functional Rating Index (n = 1, 9.1%) [47], Global Rating of Change (n = 1, 9.1%) [46], the Therapeutic Self-Care Scale (n = 1, 9.1%) [39], the Revised Illness Perception Questionnaire (n = 1, 9.1%) [39] and the Information, Autonomy and Support Scale (n = 1, 9.1%) [39].

### Reported outcomes

Mental health and HRQoL outcomes were consistently reported as being significantly impaired following an injury, including burns, blunt force trauma, road traffic crashes, poisonings, falls and drownings [39–42, 44, 46–49]. Even individuals with minor to moderate injuries showed long-term reductions in HRQoL, with many patients not returning to pre-injury health levels until six to 24 months post-injury [39–41].

Self-reported physical health was shown to improve over time, particularly between one and six months post injury, but often remained below that of non-injured people [39, 40]. In contrast, mental health recovery was slower, with persistent symptoms such as sleep disruption, psychological distress and post-traumatic stress disorder (PTSD) still reported at 12–24 months post injury, reported in 63.6% of studies (n = 7) [40, 41, 43, 46–49]. Psychosocial factors, including self-efficacy, perceived social support and illness perception, were strongly associated with recovery, particularly for patients who spent time in an Intensive Care Unit in at least one study [42]. Return to work was influenced by social functioning and employment status [43, 45] and those who made road traffic compensation claims reported negative mental and physical health outcomes, with higher rates of depression, PTSD and disability at 12 months post-injury in two studies [46, 47]. Specific to burns injuries, poor mental health, HRQoL, PTSD, self-esteem, body image and sexuality prolonged injury recovery [44, 45, 48, 49] and contributed to the time taken to returning to work in five studies (45.5%) [43, 45]. Successful access to compensation following a road traffic crash was associated with worse mental health outcomes for injured participants in two studies [46, 47]. Intentional injury, pre-existing mental health diagnoses, financial stress and housing instability were consistently associated with PROM outcomes in two studies [48, 49].

### Indigenous knowledges using the CREATE tool [33]

Out of the 11 studies, two [39, 48] (18%) included Aboriginal and Torres Strait Islander status in their study cohort. Neither reported specifically on Aboriginal or Torres Strait Islander outcomes (Table 3). Relevant Aboriginal Human Research Ethics Committee approval was not reported. Reporting on ethnicity data were conducted in demographic tables, with no further analyses or critical discussion undertaken in studies. No studies met any of the 14 CREATE criteria. No studies reported on engagement or consultation with key stakeholders or on the research teams standpoints.

### Risk of bias

An overall low risk of bias was determined for this review, with two of the 11 studies showing potential significant risk [44, 45]. Forms of bias included participant exclusion bias (i.e. disability or dominant language requirement) [44, 45, 48, 49], attrition [39–46, 48, 49], intention-to-treat analysis [42, 43, 45], appropriate analytic outcomes [44, 45], interpretation [44, 45], and presentation and reporting [45].

### Health equity using PROGRESS-PLUS [30, 34, 35]

Across the 11 studies, the most common equity variables collected were age and gender or sex (n = 11, 100%) with only one study not reporting gender [49]. Only two studies [48, 49] (18.9%) collected data across multiple PROGRESS-PLUS domains including place of residence, socioeconomic factors, race, language, occupation and education, in these studies disability was excluded along with other vulnerable groups and sexual orientation. Over half of the studies (n = 6, 55%) analysed gender in relation to outcomes with significant variability in female participation across studies (mean 26%, min = 4% [45], max = 61% [47]) (Table 3). A total of three studies associated female gender with poorer health outcomes, including poorer mental health [39], lower HRQoL [48] and negative impacts on body image and sexuality post injury [44]. In contrast, two studies reported that women had differing outcomes to men following injury, but provided no follow up discussion [43, 47], while another found that men had poorer recovery outcomes following road traffic injuries and were less likely to seek help [46]. Gender imbalance was cited as a limitation in one study [42]. while another four included gender in their analysis they did not comment on its influence [40–42, 45]. No studies used gender-inclusive language or collected data beyond the binary categories of male and female with no reporting on non-binary, gender-diverse or intersex participants.

Of the three studies (27%) [40, 46, 48] which reported on the *R* Progress criteria (1. race, 2. ethnicity, 3. culture and 4. language), none of the studies met all four criteria. One study reported both ethnicity (Aboriginal and/or Torres Strait Islander) and language (non-English speaking) [48], one reported Aboriginal and/or Torres Strait Islander status but excluded language (unable to communicate in English) [39], and one included language (non-English speaking) [46]. Three studies (27%) did not report on ethnicity or language [39, 41, 44], another four studies (36%) excluded participants unable to communicate in English [42, 43, 45, 47]. Cultural identity, including Aboriginal and Torres Strait Islander status, was limited, with two studies mentioning Aboriginal and/or Torres Strait Islanderstatus without any further analysis [39, 48], and one study including participants born overseas but provided no additional details around cultural background [48].

Disability-based exclusions (e.g. cognitive impairment, spinal cord injury or psychiatric illness) [39–43, 45, 46, 48, 49] and other vulnerable groups (e.g. incarcerated, migrants and pregnant participants) [39–42, 45–49] were common, appearing in nine studies (82%). No studies reported religion or sexual orientation. Socioeconomic status (SES) was reported in most studies (n = 8, 82%) [39–42, 46–49] with income as an indicator. Of these eight, six recorded socioeconomic factors but did not include this data in reporting their findings [39, 40, 42, 46–48] with only two discussing the role of SES in the recovery process [41, 49]. The three studies that looked at compensation following an injury did not report on SES [43, 46, 47], with one study not measuring participants SES [43] despite noting that lack of compensation can lead to an earlier return to work due to financial stress [43]. All three studies reported compensation was associated with poorer psychological outcomes and delayed recovery [43, 46, 47]. Education was reported in 64% (n = 7) [39–41, 43, 46–48] of studies with no critical analyses surrounding the impact on patient outcomes or recovery. Geographic location of residence was recorded in one study [48] but was not reported in the results or discussion.

Personal demographic information was collected in all studies, and eight demonstrated links between demographic variables and PROMs and trauma recovery. Six studies reported gender [39, 43, 44, 46–48], four studies reported age [39, 43, 48, 49], two studies reported drug use [48, 49] and income [41, 49] and one study reported social relationships [43], pre-injury mental health [49] and stable housing [49] as predictors to poorer injury recovery. While some studies demonstrated links between demographic variables and functional or mental health outcomes [39, 41, 43, 44, 46–49], there was limited equity analysis in study discussions or conclusions, and no studies fully addressed equity considerations across the PROGRESS-Plus domains with their conclusions generally lacking explicit equity framing or implications.

## Discussion

The review identified the most commonly used PROMs in observational trauma and injury studies in Australia, evaluating how, or if, health equity and Indigenous knowledges were considered or addressed. This is, to our knowledge, the first systematic review to examine PROMs following traumatic injury in an adult (≥ 18 years) cohort in Australia through an equity and Indigenous knowledge lens.

It was consistently reported that physical and mental health outcomes were significantly impaired following an injury with long term persistence into participants everyday lives [39–42, 44, 46–49]. It is well established in the literature that poorer outcomes are reported post injury across priority populations: living in regional and remote Australia [50–53], identify as Aboriginal and/or Torres Strait Islander [8, 14, 54, 55], have a pre-existing disability [56, 57], from culturally and linguistically diverse communities [58–61], or have socioeconomic disadvantage [56]. Despite this, most of these priority populations were excluded, not identified or not discussed in the majority of studies in our review. While the included studies were located in metropolitan regions, due to the Australian health care system, patients with serious injury, regardless of their background, rurality or personal circumstances are treated in large metropolitan hospitals [62].

None of the reviewed studies met all PROGRESS-PLUS standards for reporting equity-sensitive outcomes, and equity considerations were largely absent from the interpretation of findings. Where equity-relevant characteristics such as age, gender or occupation were collected, they were often used solely for descriptive purposes or controlled for in models without discussion of how they may influence outcomes. This limited the potential to understand how injury and recovery experiences vary across different sociodemographic groups, particularly for Aboriginal and Torres Strait Islander people, CALD communities, and people with lower socioeconomic status. Three studies (27%) did not report on ethnicity or language [39, 41, 44], another four studies (36%) excluded participants unable to communicate in English [42, 43, 45, 47], effectively limiting the participation of CALD populations. This has all been shown previously with current literature discussing that people from CALD backgrounds often have worse outcomes after injury [8, 14, 50–57, 63]. This can be attributed to language barriers that complicate access to culturally responsive care, due to different knowledge systems and patient fear of discrimination [58].

Gender and disability were inconsistently addressed, with notable limitations in representation and analysis. Studies had an over representation of male sex, with nine studies having over 72% of males (two studies having under but both over 45%) [39, 47]. While male sex is disproportionately burdened by injury [64], an under under-analyses of gender (i.e. male, female, LGBTQI + , non-binary) across the literature, can influence injury prevention, policy and support initiatives, contributing to a widening of the health inequity gradient across gender. Disability was often an exclusion factor which has previously been reported internationally in children [65] and is increasingly recognised as a broader issue in research. People with disabilities are often excluded from clinical trials, including those related to injury and trauma, limiting the generalisability of findings and contributing to ongoing health inequities [66, 67].

Despite Aboriginal and Torres Strait Islander communities being disproportionately burdened by injury, all studies opted to not analyse this further reflecting deep inequities in the trauma and injury research system. None of the included studies met any of the CREATE criteria, showing a lack of Aboriginal and Torres Strait Islander perspectives, values and priorities, thereby producing research that is not culturally appropriate and outcomes which perpetuate knowledge gap widening. It also emphasises the lack of current Indigenous leadership, community engagement, respect for cultural knowledge and potential for positive outcomes for Aboriginal and Torres Strait Islander in the design and use of PROMs. Aboriginal and Torres Strait Islander status was only recorded in two studies without further analysis, and neither studies reported obtaining the required Aboriginal and Torres Strait Islander ethics approval [39, 48].

Our outcomes on Indigenous knowledges and community engagement were profound, reflecting deep inequities in injury and trauma research and a lack of engagement with best practice national guidelines from the NHMRC regarding community and consumer engagement and research with Aboriginal and Torres Strait Islander communities [4, 21, 23, 24]. The exclusion of Aboriginal and Torres Strait Islander people, leaderships and knowledge in injury research limit the relevance and utility of current PROMs in measuring meaningful outcomes post injury as they fail to capture culturally significant aspects of recovery such as connection to Country, community roles and spiritual wellbeing [26]. Similar research in chronic conditions has emphasised that Aboriginal people with diabetes felt the PROMs they used did not capture their healthcare journey accurately [68]. This has contributed to ongoing policy gaps, underfunding and limited support [69, 70], further widening the health gap between injured Aboriginal and Torres Strait Islander peoples and their non-Indigenous counterparts [71].

In Australia, only two studies have developed HRQoL PROMs specific for Aboriginal and Torres Strait communities [72, 73]. Smith [72] identified twelve factors and developed them into key questions for the *Good Spirit, Good Life* tool, which provides a culturally-informed approach to measuring, understanding and meeting the quality of life needs of older Aboriginal people [74]. Howard [73] created *What Matters to Adults (WM2A)*, a QoL tool that includes 32 items across 10 domains (Balance & control; Hope & resilience; Caring for others; Culture & Country; Spirit & identity; Feeling valued; Connection with others; Access; Racism & worries; Pride & strength) [73]. The development of these two tools centred Aboriginal and Torres Strait Islander knowledges, values and leadership by having governance, Aboriginal and Torres Strait Islander led analysis, participatory research methods and continuous reflexion setting an example for future PROM development across areas of patient care and research. Incorporating culturally tailored PROMs that embed social and emotional wellbeing frameworks and address language, cultural safety and access barriers is critical to improving equity in trauma care outcomes [75].

### Strengths and limitations

This was a comprehensive systematic review collecting literature published over 25 years of prospective cohort studies investigating PROMs following a traumatic injury in Australian adults. A strength of this review was that it enabled the identification of key trends and persistent gaps, particularly the widespread exclusion of Aboriginal and Torres Strait Islander populations in adult injury research and specific PROM assessment strategies. Findings from this work will inform the development of culturally specific subsections within a well-established PROM, co-designed by and for Aboriginal and Torres Strait Islander communities. The review had its limitations as the search was limited to Australian, peer-reviewed, English-language publications and did not include studies focused on other First Nations populations or non-trauma-related health conditions such as chronic disease. While the search strategy was systematic and clearly defined, it is possible that some relevant studies were missed. The CREATE tool to assess the included studies was used by a non-Indigenous researcher (GS) which may introduce bias, however, the senior author (CR) provided regular supervision to ensure accuracy of understanding and adherence.

Nonetheless, the insights gained offer a valuable foundation for future research aimed at creating culturally safe and inclusive PROMs and underscore the need for trauma research that meaningfully incorporates Aboriginal and Torres Strait Islander voices, leadership and knowledge systems.

## Conclusion

This systematic review highlights significant gaps in the use and reporting of PROMs in Australian trauma research, particularly regarding equity and inclusion of Aboriginal and Torres Strait Islander perspectives. The consistent exclusion of priority populations, including Aboriginal and Torres Strait Islander peoples, people with disabilities, women and CALD communities, in trauma research limits the relevance and generalisability of current PROMs and risks reinforcing systemic health inequities. Addressing these gaps demands a shift toward culturally safe, inclusive and equity-sensitive PROM development and implementation. Future research must prioritise Indigenous leadership, community engagement and the integration of Indigenous knowledges to ensure PROMs reflect diverse experiences of recovery and contribute meaningfully to improved trauma care outcomes.

## Supplementary Information

Below is the link to the electronic supplementary material.

Supplementary Material 2

Supplementary Material 1

## Data Availability

No datasets were generated or analysed during the current study.

## Footnotes

1 Country—a term used commonly in Aboriginal and Torres Strait Islander communities are refers to homelands.

## References

[1] Food and Drug Administration. (2009). *Patient-Reported Outcome Measures: Use in Medical Product Development to Support Labeling Claims. Guidance for Industry*. 10.1186/1477-7525-4-79 PMC1629006 17034633

[2] Mercieca-Bebber, R., King, M. T., Calvert, M. J., Stockler, M. R., & Friedlander, M. (2018). The importance of patient-reported outcomes in clinical trials and strategies for future optimization. *Patient Related Outcome Measures,**9*, 353–367. 10.2147/prom.S156279 30464666 PMC6219423

[3] Weldring, T., & Smith, S. M. (2013). Patient-reported outcomes (PROs) and patient-reported outcome measures (PROMs). *Health Services Insights,**6*, 61–68. 10.4137/hsi.S11093 25114561 PMC4089835

[4] Ryder, C., et al. (2022). Community engagement and psychometric methods in Aboriginal and Torres Strait Islander patient-reported outcome measures and survey—A scoping review and critical analysis. *International Journal of Environmental Research and Public Health,**19*(16), Article 10354. 36011989 10.3390/ijerph191610354 PMC9407920

[5] Ryder, C., Mackean, T., Hunter, K., Towers, K., Rogers, K., Holland, A. J. A., & Ivers, R. (2020). Factors contributing to longer length of stay in Aboriginal and Torres Strait Islander children hospitalised for burn injury. *Injury Epidemiology,**7*(1), Article 52. 10.1186/s40621-020-00278-7 33012291 PMC7534159

[6] Ryder, C., & D'Angelo, S. (2022). OOPHE Impacts on SA Aboriginal Families (Community Yarns). In. Internal: Flinders University.

[7] Ryder, C., Mackean, T., Hunter, K., Rogers, K., Holland, A. J. A., & Ivers, R. (2021). Burn injuries in hospitalized Australian children—An epidemiological profile. *Journal of Burn Care & Research,**42*(3), 381–389. 10.1093/jbcr/iraa159 32910200

[8] Ryder, C., et al. (2025). “I didn’t know nothing”—Yarning up on access to compensation from road traffic injury with Aboriginal people. *Journal of Transport & Health,**42*, Article 102055. 10.1016/j.jth.2025.102055

[9] Australian Institute of Health and Welfare. (2024). Aboriginal and Torres Strait Islander Health Performance Framework: summary report August 2024. Retrieved 12/08/2024, from https://www.indigenoushpf.gov.au/getattachment/79e5f9c5-f5b9-4a1f-8df6-187f267f6817/hpf_summary-report-aug-2024.pdf

[10] Australian Institute of Health and Welfare. (2023). *Aboriginal and Torres Strait Islander Health Performance Framework: Summary report July 2023*. A. Government. https://www.indigenoushpf.gov.au/Report-overview/Overview/Summary-Report

[11] Australian Institute of Health and Welfare. (2023). *Injury in Australia*. A. Government. https://www.aihw.gov.au/reports/injury/injury-in-australia

[12] Australian Institute of Health and Welfare. (2024). *Injuries in children and adolescents 2021–22*. A. Government. https://www.aihw.gov.au/reports/injury/injuries-in-children-and-adolescents-2021-22

[13] Gabbe, B. J., Harrison, J. E., Lyons, R. A., & Jolley, D. (2011). Modelling long term disability following injury: Comparison of three approaches for handling multiple injuries. *PLoS ONE,**6*(9), Article e25862. 21984951 10.1371/journal.pone.0025862 PMC3184172

[14] Ryder, C., Mackean, T., Hunter, K., Coombes, J., Holland, A. J. A., & Ivers, R. (2021). Yarning up about out-of-pocket healthcare expenditure in burns with Aboriginal families. *Australian and New Zealand Journal of Public Health,**45*(2), 138–142. 33683766 10.1111/1753-6405.13083

[15] Australian Institute of Health and Welfare. (2024, 2024/03/21). *Self-discharge from hospital—Indigenous*. Australian Government. https://www.indigenoushpf.gov.au/measures/3-09-self-discharge-from-hospital

[16] Askew, D. A., Foley, W., Kirk, C., & Williamson, D. (2021). “I’m outta here!”: A qualitative investigation into why Aboriginal and non-Aboriginal people self-discharge from hospital. *BMC Health Services Research,**21*(1), Article 907. 10.1186/s12913-021-06880-9 34479571 PMC8414851

[17] Ryder, C., et al. (2026). Developing a patient reported measure on out-of-pocket healthcare expenditure among Aboriginal patients: A formative study. *Health Promotion International*. 10.1093/heapro/daag058 PMC13143015 42085664

[18] Ryder, C., Mackean, T., Ullah, S., Burton, H., Halls, H., McDermott, D., & Edmondson, W. (2017). Development and validation of a questionnaire to measure attitude change in health professionals after completion of an Aboriginal health and cultural safety training programme. *The Australian Journal of Indigenous Education,**48*(1), 1–15.

[19] Ryder, C., Mackean, T., Coombs, J., Hunter, K., Rogers, K., Essue, B., Holland, A. J. A., & Ivers, R. (2021). Developing economic measures for aboriginal and torres strait islander families on out-of-pocket healthcare expenditure. *Australian Healthcare Review,**45*(3), 265–273. 10.1071/AH20299 34016253

[20] Le Grande M, S. C., Thompson, D. R., Scuffham P., Kularatna S., Jackson A. C., Brown A. (2017). Social and emotional wellbeing assessment instruments for use with Indigenous Australians: A critical review. *Social Science Medicine*, *187*, 164–173. 10.1016/j.socscimed.2017.06.046 28689090

[21] National Health and Medical Research Council. (2018). *Keeping research on track II: A companion document to Ethical conduct in research with Aboriginal and Torres Strait Islander peoples and communities: Guidelines for researchers and stakeholders*. Commonwealth of Australia.

[22] Vogt, D. S., King, D. W., & King, L. A. (2004). Focus groups in psychological assessment: Enhancing content validity by consulting members of the target population. *Psychological Assessment,**16*(3), 231–243. 10.1037/1040-3590.16.3.231 15456379

[23] Australian Institute of Aboriginal and Torres Strait Islander Studies. (2020). *AIATSIS code of ethics for Aboriginal and Torres Strait Islander research*. Australian Institute of Aboriginal and Torres Strait Islander Studies.

[24] National Health and Medical Research Council. (2018). *Road map 3: A strategic framework for improving Aboriginal and Torres Strait Islander health through research*. Commonwealth of Australia. 10.5694/j.1326-5377.2008.tb01767.x 18459924

[25] Kite, E., Davy, C., Gibson, O., Brown, K., & Brown, A. (2014). Aboriginal and Torres Strait Islander peoples’ perceptions of quality of life and wellbeing and how they are measured: A systematic review protocol. *JBI Database of Systematic Reviews and Implementation Reports,**12*, 138. 10.11124/jbisrir-2014-1529

[26] Kite, E., & Davy, C. (2016). Using Indigenist and Indigenous methodologies to connect to deeper understandings of Aboriginal and Torres Strait Islander peoples’ quality of life. *Health Promotion Journal of Australia,**26*(3), 191–194. 10.1071/HE15064 26686300

[27] Schamber, E. M., Takemoto, S. K., Chenok, K. E., & Bozic, K. J. (2013). Barriers to completion of patient reported outcome measures. *The Journal of Arthroplasty,**28*(9), 1449–1453. 10.1016/j.arth.2013.06.025 23890831

[28] Lyons, R. A., et al. (2007). The UK burden of injury study—A protocol. [National Research Register number: M0044160889]. *BMC Public Health*,* 7*, 317. 10.1186/1471-2458-7-317 PMC2225415 17996057

[29] Van Beeck, E. F., Larsen, C. F., Lyons, R. A., Meerding, W. J., Mulder, S., & Essink-Bot, M. L. (2007). Guidelines for the conduction of follow-up studies measuring injury-related disability. *Journal of Trauma,**62*(2), 534–550. 10.1097/TA.0b013e31802e70c7 17297349

[30] Welch, V., Petticrew, M., Tugwell, P., Moher, D., O’Neill, J., Waters, E., & White, H. (2012). PRISMA-Equity 2012 extension: Reporting guidelines for systematic reviews with a focus on health equity. *PLoS Medicine,**9*(10), Article e1001333. 10.1371/journal.pmed.1001333 23222917 PMC3484052

[31] Huria, T., Palmer, S. C., Pitama, S., Beckert, L., Lacey, C., Ewen, S., & Smith, L. T. (2019). Consolidated criteria for strengthening reporting of health research involving indigenous peoples: The CONSIDER statement. *BMC Medical Research Methodology,**19*(1), 173. 10.1186/s12874-019-0815-8 31399058 PMC6688310

[32] Harfield, S., et al. (2018). *The Aboriginal and Torres Strait Islander Quality Appraisal Tool: Companion Document*.

[33] Harfield, S., et al. (2020). Assessing the quality of health research from an Indigenous perspective: The Aboriginal and Torres Strait Islander quality appraisal tool. *BMC Medical Research Methodology,**20*(1), 79. 10.1186/s12874-020-00959-3 32276606 PMC7147059

[34] Kavanagh, J., Oliver, S., & Lorenc, T. (2008). Reflections on developing and using PROGRESS-Plus. *Equity Update,**2*, 1–3.

[35] Waters, E., de Silva-Sanigorski, A., Hall, B. J., Brown, T., Campbell, K. J., Gao, Y., Armstrong, R., Prosser, L., & Summerbell, C. D. (2011). Interventions for preventing obesity in children. *Cochrane Database System Review 12*, Cd001871. 10.1002/14651858.CD001871.pub3 22161367

[36] Popay, J., Roberts, H., Sowden, A., Petticrew, M., Arai, L., Rodgers, M., Britten, N., Roen, K., & Duffy, S. (2006). *Guidance on the conduct of narrative synthesis in systematic reviews: A product from the ESRC Methods Programme*. 10.13140/2.1.1018.4643

[37] Petticrew, M., & Roberts, H. (2006). *Systematic Reviews in the Social Sciences: A Practical Guide* (Vol. 11). 10.1002/9780470754887

[38] Viswanathan, M., & Berkman, N. D. (2012). Development of the RTI item bank on risk of bias and precision of observational studies. *Journal of Clinical Epidemiology,**65*(2), 163–178. 10.1016/j.jclinepi.2011.05.008 21959223

[39] Aitken, L. M., Chaboyer, W., Kendall, E., & Burmeister, E. (2012). Health status after traumatic injury. *Journal of Trauma and Acute Care Surgery,**72*(6), 1702–1708. 10.1097/TA.0b013e318246bfe9 22695444

[40] Aitken, L. M., Chaboyer, W., Schuetz, M., Joyce, C., & Macfarlane, B. (2014). Health status of critically ill trauma patients. *Journal of Clinical Nursing,**23*(5–6), 704–715. 10.1111/jocn.12026 23228039

[41] Aitken, L. M., Macfarlane, B., Chaboyer, W., Schuetz, M., Joyce, C., & Barnett, A. G. (2016). Physical function and mental health in trauma intensive care patients: A 2-year cohort study. *Critical Care Medicine,**44*(4), 734–746. 10.1097/ccm.0000000000001481 26646456

[42] Connolly, F. R., Aitken, L. M., Tower, M., & Macfarlane, B. (2014). Factors associated with self-efficacy for managing recovery in the trauma intensive care population: A prospective cohort study. *Injury,**45*(1), 272–278. 10.1016/j.injury.2013.05.005 23747123

[43] Clay, F. J., Fitzharris, M., Kerr, E., McClure, R. J., & Watson, W. L. (2012). The association of social functioning, social relationships and the receipt of compensation with time to return to work following unintentional injuries to Victorian workers. *Journal of Occupational Rehabilitation,**22*(3), 363–375. 10.1007/s10926-012-9354-4 22278297

[44] Connell, K. M., Coates, R., & Wood, F. M. (2013). Sexuality following burn injuries: A preliminary study. *Journal of Burn Care & Research,**34*(5), e282-289. 10.1097/BCR.0b013e31827819bf 23377351

[45] Dowda, D. J., & Li, F. (2014). Major concerns and issues in burn survivors in Australia. *Burns & Trauma,**2*(2), 84–87. 10.4103/2321-3868.130192 27602366 PMC5012066

[46] Murgatroyd, D. F., Harris, I. A., Tran, Y., & Cameron, I. D. (2016). The association between seeking financial compensation and injury recovery following motor vehicle related orthopaedic trauma. *BMC Musculoskeletal Disorders,**17*, Article 282. 10.1186/s12891-016-1152-2 27411446 PMC4944484

[47] Littleton, S. M., Cameron, I. D., Poustie, S. J., Hughes, D. C., Robinson, B. J., Neeman, T., & Smith, P. N. (2011). The association of compensation on longer term health status for people with musculoskeletal injuries following road traffic crashes: Emergency department inception cohort study. *Injury,**42*(9), 927–933. 10.1016/j.injury.2010.02.011 22081822

[48] Druery, M., Das, A., Warren, J., Newcombe, P. A., Lipman, J., & Cameron, C. M. (2024). Early predictors of health-related quality of life outcomes at 12 months post-burn: ABLE study. *Injury,**55*(6), Article 111545. 10.1016/j.injury.2024.111545 38584078

[49] Ford, D., Waller, M., Das, A., Cameron, C. M., Warren, J., & Druery, M. (2025). Baseline predictors of depression and post-traumatic stress disorder (PTSD) symptoms in hospitalised adult burn survivors: A longitudinal, prospective cohort study. *Injury,**56*(3), Article 112151. 10.1016/j.injury.2025.112151 39883967

[50] Australian Institute of Health and Welfare. (2024). *Rural and Remote health*. A. Government. https://www.aihw.gov.au/reports/rural-remote-australians/rural-and-remote-health

[51] Read, D. J., Hayes, I., & Sullivan, S. G. (2025). The association between remoteness of injury and in-hospital mortality for motor vehicle collision major trauma patients: Evidence of survivor bias in an analysis of registry data. *Injury Epidemiology,**12*(1), Article Article 40. 10.1186/s40621-025-00586-w PMC12239381 40629480

[52] Heathcote, K., et al. (2022). Rural and urban patterns of severe injuries and hospital mortality in Australia: An analysis of the Australia New Zealand Trauma Registry: 2015–2019. *Injury,**53*(6), 1893–1903. 10.1016/j.injury.2022.03.044 35369988

[53] Australian Institute of Health and Welfare. (2021). *Geographical Analysis of Hospitalised Injury and Injury Deaths Data*. A. Government. https://www.aihw.gov.au/reports/injury/geographical-analysis-injury-data/contents/about

[54] Katzenellenbogen, J. M., et al. (2018). Missing voices: Profile, extent, and 12-month outcomes of nonfatal traumatic brain injury in Aboriginal and non-Aboriginal adults in Western Australia using linked administrative records. *Journal of Head Trauma Rehabilitation*. 10.1097/htr.0000000000000371 29601340

[55] Irie, F., Pollard, C., & Bellamy, N. (2010). Characteristics and outcomes of injury patients in an Aboriginal and Torres Strait Islander population—Queensland Trauma Registry, Australia. *Injury,**41*(9), 964–969. 10.1016/j.injury.2009.09.002 19775687

[56] Gabbe, B. J., et al. (2016). Return to work and functional outcomes after major trauma who recovers, when, and how well? *Annals of Surgery,**263*(4), 623–632. 10.1097/SLA.0000000000001564 26779977

[57] Quested, R., Sommerville, S., & Lutz, M. (2017). Outcomes following non-life-threatening orthopaedic trauma: Why are they considered to be so poor? *Trauma,**19*(2), 133–138. 10.1177/1460408616674233

[58] Le, K. D. R., Le, K., Shahzad, A., & Lee, S. J. (2024). The role of language barriers on hospital outcomes in culturally and linguistically diverse patients following trauma admission. *Trauma Care,**4*(2), 107–119.

[59] Saltapidas, H., & Ponsford, J. (2008). The influence of cultural background on experiences and beliefs about traumatic brain injury and their association with outcome. *Brain Impairment,**9*(1), 1–13. 10.1375/brim.9.1.1

[60] Clark, A., Gilbert, A., Rao, D., & Kerr, L. (2014). “Excuse me, do any of you ladies speak English?” Perspectives of refugee women living in South Australia: Barriers to accessing primary health care and achieving the quality use of medicines. *Australian Journal of Primary Health,**20*(1), 92–97. 10.1071/py11118 23482062

[61] Saltapidas, H., & Ponsford, J. (2007). The influence of cultural background on motivation for and participation in rehabilitation and outcome following traumatic brain injury. *The Journal of Head Trauma Rehabilitation,**22*(2), 132–139. 10.1097/01.HTR.0000265101.75177.8d 17414315

[62] Warren, K.-R.J., Morrey, C., Oppy, A., Pirpiris, M., & Balogh, Z. J. (2019). The overview of the Australian trauma system. *OTA International: The Open Access Journal of Orthopaedic Trauma,**2*(S1), e018–e018. 10.1097/OI9.0000000000000018 37675256 PMC10479347

[63] Gabbe, B. J., Cameron, P. A., Williamson, O. D., Edwards, E. R., Graves, S. E., & Richardson, M. D. (2007). The relationship between compensable status and long‐term patient outcomes following orthopaedic trauma. *Medical Journal of Australia,**187*(1), 14–17. 10.5694/j.1326-5377.2007.tb01108.x 17605697

[64] Health, A. I. o., & Welfare. (2024). *Injuries affecting men in Australia: A closer look*. https://www.aihw.gov.au/reports/injury/injuries-affecting-men-in-australia-a-closer-look

[65] Ryder, C., Mackean, T., Hunter, K., Williams, H., Clapham, K., Holland, A. J. A., & Ivers, R. (2020). Equity in functional and health related quality of life outcomes following injury in children—A systematic review. *Critical Public Health,**30*(3), 352–366. 10.1080/09581596.2019.1581918

[66] Shariq, S., Cardoso Pinto, A. M., Budhathoki, S. S., Miller, M., & Cro, S. (2023). Barriers and facilitators to the recruitment of disabled people to clinical trials: A scoping review. *Trials,**24*(1), Article 171. 10.1186/s13063-023-07142-1 36890505 PMC9994780

[67] Cunningham, S., Russell, A. M., Lidington, E., & Shiely, F. (2025). Lack of data collection in clinical trials prevents us from evaluating inclusion of people with disabilities. *Journal of Clinical Epidemiology,**181*, Article 111715. 10.1016/j.jclinepi.2025.111715 39924129

[68] Burgess, A., Hawkins, J., Kostovski, C., Kennedy, M., Penkala, S., & Duncanson, K. (2023). Aboriginal people’s perceptions of patient-reported outcome measures in the assessment of diabetes health-related quality of life. *Australian Journal of Primary Health,**29*(2), 165–174. 10.1071/py22150 37079465

[69] Australian Government Department of Health Disability and Ageing. (2021). Status and determinants of Aboriginal and Torres Strait Islander Health. https://www.health.gov.au/topics/aboriginal-and-torres-strait-islander-health/status-and-determinants

[70] Biles, B. J., Serova, N., Stanbrook, G., Brady, B., Kingsley, J., Topp, S. M., & Yashadhana, A. (2024). What is indigenous cultural health and wellbeing? A narrative review. *The Lancet Regional Health—Western Pacific,**52*, Article 101220. 10.1016/j.lanwpc.2024.101220 39664592 PMC11632815

[71] AIHW. (2022). *Determinants of health for Indigenous Australians*. https://www.aihw.gov.au/reports/australias-health/social-determinants-and-indigenous-health

[72] Smith, K., et al. (2021). Good spirit, good life: A quality of life tool and framework for older Aboriginal peoples. *The Gerontologist,**61*(5), e163–e172. 10.1093/geront/gnz185 32191314

[73] Howard, K., et al. (2024). Development of the What Matters 2 Adults (WM2A) wellbeing measure for Aboriginal and Torres Strait Islander adults. *Social Science & Medicine,**347*, Article 116694. 10.1016/j.socscimed.2024.116694 38569315

[74] Smith, K., Gilchrist, L., Bessarab, D., Taylor, K., Clinch, C., Edgill, P., Flicker, L., Hayden, S., & Petersen, C. (2023). *Good Spirit Good Life (GSGL) Assessment Tool: Client and Carer versions*. https://www.iawr.com.au/Profiles/aww/Assets/ClientData/GSGL_Instructions_28_July_2023.pdf

[75] Murray, K., Nebeker, C., & Carpendale, E. (2019). Responsibilities for ensuring inclusion and representation in research: A systems perspective to advance ethical practices. *Australian & New Zealand Journal of Psychiatry,**53*(9), 835–838. 10.1177/0004867419865334 31339346

