# Supplementary material for: Equity, evidence and indigenous knowledges in PROMs in Australian injury research: a systematic review
Source: Qual Life Res. 2026 Jul 30;35(9):250. doi: 10.1007/s11136-026-04353-2 (PMC13424147; doi:10.1007/s11136-026-04353-2)
Supplement: Supplementary file 2 — Supplementary Material 1 [file 11136_2026_4353_MOESM3_ESM.docx]

Studies from databases/registers **(n = 4120)**

Scopus (n = 1823)

MEDLINE (n = 1343)

CINAHL (n = 952)

Citation searching (n = 2)

**Identification**

Included studies ongoing **(n = 0)**

Studies awaiting classification **(n = 0)**

Studies included in review **(n = 11)**

Studies excluded **(n = 2965)**

Studies assessed for eligibility **(n = 99)**

Studies sought for retrieval **(n = 99)**

Studies screened **(n = 3064)**

Studies excluded **(n = 88)**

Wrong setting (n = 9)

Wrong outcomes (n = 6)

Excluded Country (n = 5)

Wrong study design (n = 40)

Excluded Date Range (n = 1)

Wrong Publication Type (n = 6)

Full text not avaliable (n = 1)

Wrong patient population (n = 20)

References removed **(n = 1056)**

Duplicates identified manually (n = 24)

Duplicates identified by Covidence (n = 1032)

Marked as ineligible by automation tools (n = 0)

Other reasons (n = 0)

**Screening**

**Included**
